# Single Antenatal Exposure to Ciclesonide Reduces Long-Term Cardiac Structural and Functional Alterations Compared to Currently Approved Synthetic Corticosteroids

**DOI:** 10.21203/rs.3.rs-9283462/v1

**Published:** 2026-05-18

**Authors:** Shekhar Gugnani, Tooba Fida, Rachel Tao, Keya Panchal, Julian Vallejo, Donald DeFranco, Michael Wacker, A.Paula Monaghan Nichols

**Affiliations:** University of Missouri–Kansas City; University of Missouri–Kansas City; University of Missouri–Kansas City; University of Missouri–Kansas City; University of Missouri–Kansas City; University of Pittsburgh School of Medicine; University of Missouri–Kansas City; University of Missouri–Kansas City

## Abstract

Synthetic corticosteroids (sCS), Dexamethasone (Dex) or Betamethasone (Beta) are administered to mothers at risk of preterm birth to promote fetal organ maturation and reduce neonatal morbidity. Repeated prenatal or postnatal sCS exposure is associated with long term negative cardiovascular and neurologic outcomes. We previously demonstrated that repeated postnatal exposure to Ciclesonide (Cic) promotes lung maturation and minimizes birthweight or white matter reductions observed with repeated Dex administration. The long-term cardiac effects of a single antenatal sCS exposure are poorly understood. This study demonstrates that a single prenatal exposure to sCS reduces birth weight in a graded manner, with Dex>Beta > Cic. In aged animals Dex exposure led to an increase in body weight, Beta showed a trend towards a decrease, while Cic was indistinguishable from controls. Structural, histological, and electrophysiological cardiac abnormalities consistent with bradycardia and QTc prolongation were exclusively observed in aged Dex-exposed animals. Adult cardiac ion channel expression was decreased with Dex>Beta > Cic, indicating that the antenatal environment plays a pivotal role in short-term and long-term cardiac reprogramming. A single antenatal exposure to Cic minimizes adverse cardiometabolic effects compared to Dex or Beta suggesting that Cic may be a safer alternative for preterm birth compared to the current sCS clinical regimen.

## Introduction

Preterm birth and its associated postnatal complications are one of the leading causes of death in children under the age of five, affecting more than 1 in 8 babies in the United States and almost one million babies per year globally^1–3^. Preterm birth poses significant short-term and long-term risks to both maternal and neonatal well-being^4–6^. In 2019, the rate of preterm birth in the United States rose to 10.23%, marking it the fifth consecutive year with an annual increased rate and the highest level of preterm birth in more than a decade^7–9^. The current clinically approved regimen for mothers at risk of preterm birth in the United States is the administration of synthetic corticosteroids (sCS); among these, the most widely used are Dexamethasone (Dex) and Betamethasone (Beta)^10–12^. These drugs accelerate fetal lung maturation, reducing the risk of intraventricular hemorrhage; necrotizing enterocolitis; and bronchopulmonary dysplasia, and significantly reducing neonatal death and morbidity as well as the need for intensive postnatal care^13–16^. While the short-term benefits of antenatal sCS in improving neonatal outcomes are well-established, their usage has been associated with adverse long-term systemic effects.

Antenatal corticosteroid exposure has been linked to intrauterine growth restriction (characterized by low birth weight and multi-organ system dysfunction), with negative effects often persisting beyond birth^17,18^. Considerable evidence indicates that repeated antenatal exposure to sCS can cause neurological abnormalities, including increased risk for cerebral palsy, abnormal neurologic examinations, higher Major Depression Inventory scores, and cortical white matter deficits^17,19^. One area of concern is the impact of corticosteroids on the cardiovascular system. Prolonged antenatal, perinatal and adult exposure to sCS is associated with the cardinal features of metabolic disease, such as insulin resistance, hypertension, dyslipidemia, and obesity; these factors increase the risk for cardiovascular disease and ultimately stroke, arrhythmia, or heart failure^20–22^.

Evidence in human and animal models indicates that repeated antenatal exposure to sCS leads to abnormal myocardial function, hypertension, and lasting cardiac dysfunction that is present not only at the time of birth but also throughout life^17,23–26^. Adult pregnant rats repeatedly exposed with up to 0.5 mg/kg Dex antenatally exhibit myocardial cell hypertrophy and a significant decrease in ventricular weight^17^. Antenatal sCS-induced cardiovascular dysfunction is associated with the generation of oxidative stress at birth^26^. Corticosteroids are potent inducers of reactive oxygen species (ROS), which can trigger hypertension and endothelial dysfunction (effects that can be prevented by antioxidant treatment). Therefore, mechanisms mediating off-target adverse effects of sCS may relate to the induction of excess ROS production and associated intracellular signaling, including the activation of stress kinases, cell cycle changes, and the induction of cell senescence^21,26^.

The American College of Obstetricians and Gynecologists recommends a single course of sCS for pregnant women between 23 and 36 gestation weeks who are at risk for preterm birth within 7 days^27^. Given the potential systemic and cardiac side effects of traditional sCS administration, there is an urgent need to identify the long term consequences of a single clinically relevant exposure to sCS and to identify alternative treatments that can provide similar developmental and pathophysiological benefits while minimizing adverse long-term systemic effects. Ciclesonide (Cic, ALVESCO) is a prodrug that is converted into the metabolically active form desisobutyryl-ciclesonide (Des) by endogenous carboxylesterase enzymes predominantly expressed in the liver, intestine, respiratory tract, and placenta^28–30^. Cic is currently clinically approved as an inhaled preparation for the treatment of asthma in children over the age of 12 and as a nasal spray for allergic rhinitis in children greater than 6 years of age and adults^30,31^. Cic may be a safer alternative to Dex or Beta due to its high plasma protein binding affinity (> 99%) and high pulmonary lipid affinity; the drug has rapid clearance of its active form, with limited systemic circulation of unbound fractions^32–34^. Previous studies demonstrate that Cic administration in post-natal pups activates pulmonary corticosteroid responses without leading to the adverse effects on body growth, brain weight, or white matter loss observed with Dex exposure^34,35^. This suggests that Cic could be a safer sCS therapy for prematurity, potentially limiting long-term cardiovascular effects^34^.

Although extensive research has focused on the immediate benefits and risks of repeated antenatal sCS administration, few studies address the potential long-term effects of these drugs on offspring. Published long-term studies use multiple or high dose sCS administration. Additionally, there is limited research on the underlying mechanisms and long-term consequences of a single-dose antenatal clinically relevant sCS exposure. This study compared the molecular, pathophysiological and functional consequences of a single antenatal Dex, Beta or Cic exposure on both the immediate and long-term metabolic or cardiac function. Furthermore, this study seeks to evaluate whether Cic could serve as a safer alternative for the management of preterm birth limiting cardiovascular dysfunction.

## Materials & Methodology

### Animal Protocols

All animal experiments were performed according to approved Institutional Animal Care and Use Committee protocols at the University of Missouri – Kansas City (UMKC), conforming to relevant federal guidelines. The UMKC animal facility is operated as a specific pathogen free, AAALAC accredited, PHS assured facility. Animal care and husbandry meets the requirements in the Guide for the Care and Use of Laboratory Animals (8th edition), National Research Council. Animals are group housed and maintained on a 12 hour light/dark cycle with ad libitum food and water at a constant temperature of 70–72° F and humidity of 30–70%. Daily health check inspections are performed by qualified veterinary staff and/or animal care technicians. Timed pregnant mice were purchased from Charles River, arrived at embryonic day E12, and were housed for two days in the animal facility before exposure to drugs. Males were singly housed while females were housed in groups of four. The Institutional Animal Care and Use Committee (IACUC) at the University of Missouri-Kansas City (Protocol #45543) approved all experimental animal procedures which were performed in accordance with institutional, federal, and ARRIVE guidelines. Animals were euthanized according to the 2020 American Veterinary Medical Association guidelines for CO2 asphyxiation and cervical dislocation. Males and females were randomly assigned to each treatment or control group. Sample size was determined in previous studies using a power analysis indicating that a size of 4–7 animals per group would be sufficient to achieve the expected effect size at an α of 0.05^17,19,34^.

### Drug dose and tissue collection:

Timed pregnant CD1 mice were antenatally injected with a single intraperitoneal administration of vehicle (Veh, 0.001% Ethanol in phosphate buffered saline pH 7.2 (PBS), Dex, Beta, or Cic (0.4mg/kg, Millipore Sigma) at Embryonic Day 14.5 (E14.5). This approximates the minimal dose used clinically in humans (0.35 mg/ kg) and as previously reported^19,36^. Body weight was monitored in the first week after birth (Postnatal Day 1–7, P1-P7) until 92 weeks of age. Weight curves were generated and statistically compared in GraphPad and curve intersection was calaculated using Desmos online graphing calculator. For metabolic analyses, serum glucose and cholesterol levels were measured at the end of life using a OneTouch AimStrip Tandem Lipid profile and Glucose Measuring System (Ermaine Laboratories).

### Histological analyses:

Hearts were collected at birth or adulthood and weighed. Adult hearts were immersed in cardioplegic solution (20mM KCl) and manually pumped for 30 seconds and fixed in Carnoy’s fixative (60% Ethanol, 30% Chloroform, and 10% Glacial Acetic Acid). P1 hearts were immediately fixed and not placed into cardioplegic solution due to their minute size. Hearts were embedded in paraffin and sectioned at 15 micrometers. Every 4th section was stained with Hematoxylin & Eosin (H&E) for structural analysis (N = 4–6 per treatment) and every 5th section stained with Masson’s Trichrome (N = 4–7 per treatment) for quantitative collagen analysis. The results were examined histologically using both a light microscope and an Invitrogen EVOS^™^ FL Auto 2 Imaging System by an observer blind to the treatment. For H&E-stained sections, four high-resolution images per slide at 20x magnification were taken of ventricle muscle and stitched together via Auto 2 Imaging Software. For Trichrome-stained sections, three high-resolution transverse and sagittal images per slide were taken at 20x magnification of ventricle muscle with peripheral collagen. Coronal and horizontal cross-sections were imaged for P1 and adult hearts, respectively; left and right ventricular free wall diameter, left and right ventricular chamber size, and septal thickness were digitally measured using QuPath Open-Source Software^37^. Cardiac collagen was quantified using the National Institute of Health ImageJ Open-Source Software with the Color Deconvolution 2 Plugin^38^.

### Electrocardiogram Analyses:

From 72–76 weeks of age, electrocardiogram (ECG) analysis was performed using ECGenie (MouseSpecifics) in conscious animals (N = 6–8 per treatment). Animals were placed on an elevated platform and allowed to acclimatize for 10 minutes before the collection of baseline data. Recordings were gathered over a 15-to-60-minute period until 12–15 distinct consecutive episodes of five or more identifiable QRS peaks of electrophysiological activity were recorded. Data was analyzed using e-MOUSE^™^ digital software. The cardiac heart rate (HR), RR, PR, QRS, ST, and corrected QT (QTc) intervals were recorded and averaged across all ECGs per mouse per treatment. Heart rate was measured in beats per millisecond (bpms).

### RNA Isolation and Quantitative Polymerase Chain Reaction:

Following ECG analysis, adult hearts were excised, and horizontal sections isolated from the middle widest cross-sectional area of the heart and used for RNA isolation using Trizol Extraction Reagent per the manufacturer’s instructions (Invitrogen by Thermo Fisher Scientific). RNA was converted to cDNA using ThermoFisher high-capacity RNA-to-cDNA kits, and Quantitative Polymerase Chain Reaction (qPCR) was performed using SYBR^™^ Green Master Mix with primers for key cardiac potassium and calcium ion channel genes (N = 3–5 per treatment) (Supplementary Table S1).

### Statistical Analyses:

GraphPad Prism 10 statistical software was used to calculate significance and generate graphs (GraphPad Software Inc., La Jolla, CA). Data are presented as mean ± standard error of the mean (SEM) where indicated. Differences between control and experimental groups (N = 4–12 per experimental group) were compared using the following tests: One-Way ANOVA per time period with Dunnett’s post-hoc tests (body weight, ECG analyses, and PCR analyses), Log-Rank Mantel-Cox test (survival), and unpaired T-Tests (heart dimensions and collagen quantification). Mean and standard error of the mean were calculated for body weight and collagen quantification. To identify outliers, a Grubbs Outlier Test with an α of 0.05 was used. Additionally, a Welch’s One-Way ANOVA was used when Bartlett’s test of homogeneity indicated unequal variances (heart rate in ECG analyses).

## Results

### A single antenatal exposure to Dex, Beta, or Cic leads to distinct birth weight and growth trajectory:

**Figure 1:**
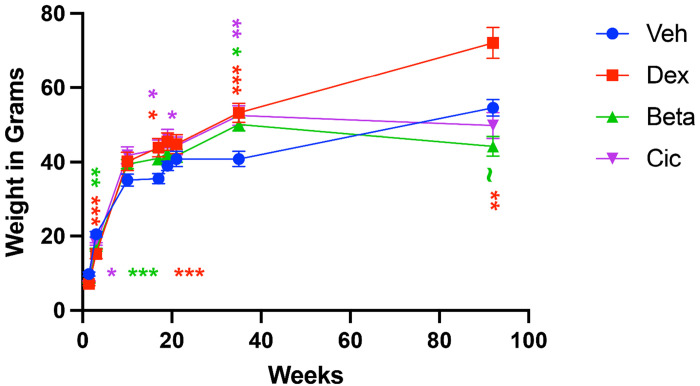
Growth Curves in Mice Prenatally Exposed to Distinct sCS. Growth Curves from birth to end of life (~ 92 weeks) of sCS-exposed CD1 mice. Prenatal exposure to a single dose of any sCS (Dex, Beta, or Cic) led to a significant decrease in birth weights compared to control (Dex: p = 0.0003; Beta: p = 0.0008; Cic: p = 0.0116), but by week 10 this difference was no longer statistically significant. After 35 weeks, Dex-, Beta-, and Cic-treated mice showed an increase in weight compared to controls. By week 92, Dex animals were significantly obese (72g) compared to controls (54.57g, p = 0.0023). Beta trended toward a decrease in body weight (44.2g, “~” p-value of 0.0509), while Cic demonstrated no significant difference (49.8g, p = 0.5291). Significance values: * = p < 0.05, ** = p < 0.01, *** = p < 0.001.

It is well-established in both rodent and human studies that sCS administration for the management of prematurity results in a significant decrease in birth weight^19,39–42^. From birth to week one, in utero exposure to Dex, Beta, or Cic led to a graded reduction in birth weight compared to controls (Dex 34% decrease: p = 0.0003; Beta 31% decrease: p = 0.0008; Cic 26% decrease: p = 0.0116) (Supplemental Table S2). By postnatal day 7 (week 1), body weights remained significantly lower in Dex- (25%, p = 0.0008) and Beta-treated groups (19%, p = 0.0044), Cic-exposed animals were indistibguishable from controls (p = 0.1126). By week 5, weights were similar to controls in all treatment groups. Between weeks 10 and 17, significant elevation in body weights became apparent in Dex and Cic treatment groups relative to controls (Dex: p = 0.0189; Cic: p = 0.0332). At the end of life (92 weeks), Dex animals maintained significantly higher body weights compared to Veh (Dex 72g, Veh 54.57; 32% increase; p = 0.0023). Beta-treated animals showed a trend towards a decrease (44.2g; 19% decrease; p = 0.0509), while Cic-treated animals showed no significant difference (49.8g; p = 0.5291). The increase in weight in Dex exposed animals was due to an observed increase in abdominal fat deposition in Dex versus controls. These findings demonstrate a biphasic growth response: early postnatal growth suppression followed by progressive weight gain in adulthood, which is particularly pronounced in the Dex group (Fig. 1).

### Blood glucose and cholesterol levels are normal in experimental versus control groups:

Previous studies in humans have shown that antenatal sCS administration is associated with an increased risk for metabolic disease in adulthood, characterized by obesity, insulin resistance, hypertension, and cardiovascular disease^18,41,43–45^. Considering the increased weight in aged Dex- treated animals, we measured blood glucose and cholesterol levels in adults. Blood glucose concentrations were not significantly different between experimental and control free-eating groups: Veh (9.6 mmol/L), Dex (9.0 mmol/L), Beta (9.0 mmol/L), and Cic (11.0 mmol/L) (N = 5–7 per treatment; Dex: p = 0.82; Beta p = 0.38, Cic p = 0.17). Additionally, cholesterol levels remained within the normal physiological range for all groups, indicating that single-dose antenatal sCS exposure did not result in notable metabolic disturbances under the conditions tested.

### Antenatal exposure to sCS does not alter long-term survival:

**Figure 2:**
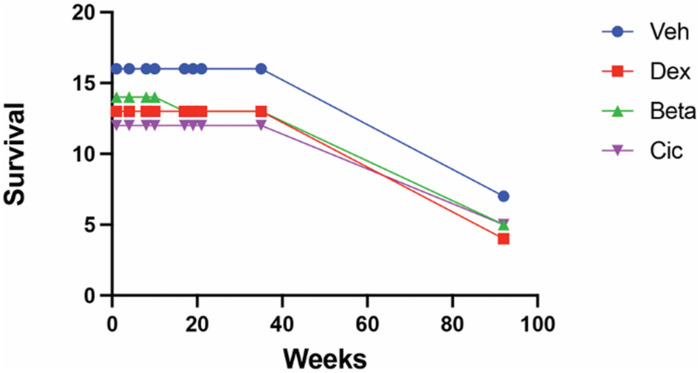
Survival Curve Comparisons in Mice Prenatally Exposed to Distinct sCS. Survival Curves from birth to end of life (92 weeks) in sCS-exposed CD1 mice. Mantel-Cox Log-Rank test revealed no significant observed difference in survival between treated groups. At birth, N=Veh 16, Dex 13, Beta 13, Cic 12. At end of life, N=Veh 7, Dex 4, Beta 5, Cic 5.

To determine if antenatal exposure to sCS altered longevity, survival rates of the animals were monitored over a 92 week period. From birth to 92 weeks no significant difference in survival was observed (p = 0.64 for all groups). At birth, the number of animals in each group was: Veh (N = 16), Dex (N = 13), Beta (N = 13), and Cic (N = 12); by 92 weeks, the number of surviving animals in each group was: Veh (N = 7), Dex (N = 4), Beta (N = 5), and Cic (N = 5) (Fig. 2). When separated by sex, no significant differences in survival were observed between treatment groups. These findings suggest that antenatal exposure to sCS does not significantly impact long-term survival, despite the observed physiological and cardiac alterations described below.

### Antenatal Dex exposure leads to cardiac pathology from birth to adulthood:

**Figure 3:**
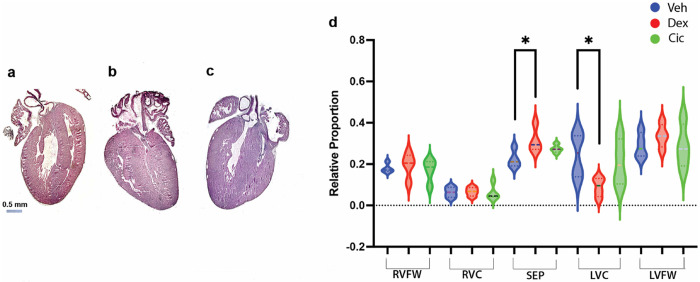
Histological and Structural Cardiac Alterations of P1 Mice Prenatally Exposed to Distinct sCS. a,b,c: Hematoxylin & Eosin-stained 4x coronal images of P1 hearts exposed to (a) Veh, (b), Dex, or (c) Cic at birth. d: Violin plots of Right Ventricular Free Wall (RVFW), Right Ventricular Chamber (RVC), Septum, Left Ventricular Chamber (LVC), and Left Ventricular Free Wall (LVFW) measurement comparisons on postnatal day 1. Left ventricular chamber size decreased by 64% and septum size increased by 41% in Dex-exposed mice compared to controls at birth (N=4; p = 0.042, p = 0.031 respectively).

To evaluate potential short-term cardiac effects of antenatal sCS exposure, hearts were examined at postnatal day 1 (P1). In light of the observed significant differences in birth weight and growth trajectory in Dex-exposed compared to Cic-exposed, these studies focused on Dex versus Cic comparisons. Previous studies have indicated that while repeated antenatal sCS exposure stimulates cardiomyocyte proliferation and energy production, adverse side effects are also observed leading to cardiac alterations at birth^41,46–48^. Few studies have investigated the consequences of a single physiologically relevant sCS on the neonatal heart. To examine the effects of sCS exposure on the heart’s anatomical structure at P1, we measured wall thicknesses and ventricular diameters of both the left and right ventricles. At birth, the left ventricle chamber size in Dex-exposed animals was decreased by 64% (p = 0.042), while the septal thickness increased by 41% compared to Veh (N = 4; p = 0.031). These structural changes were not observed in Cic-exposed animals (N = 4; left ventricle chamber: p = 0.662; septum: p = 0.231, Fig. 3).

**Figure 4:**
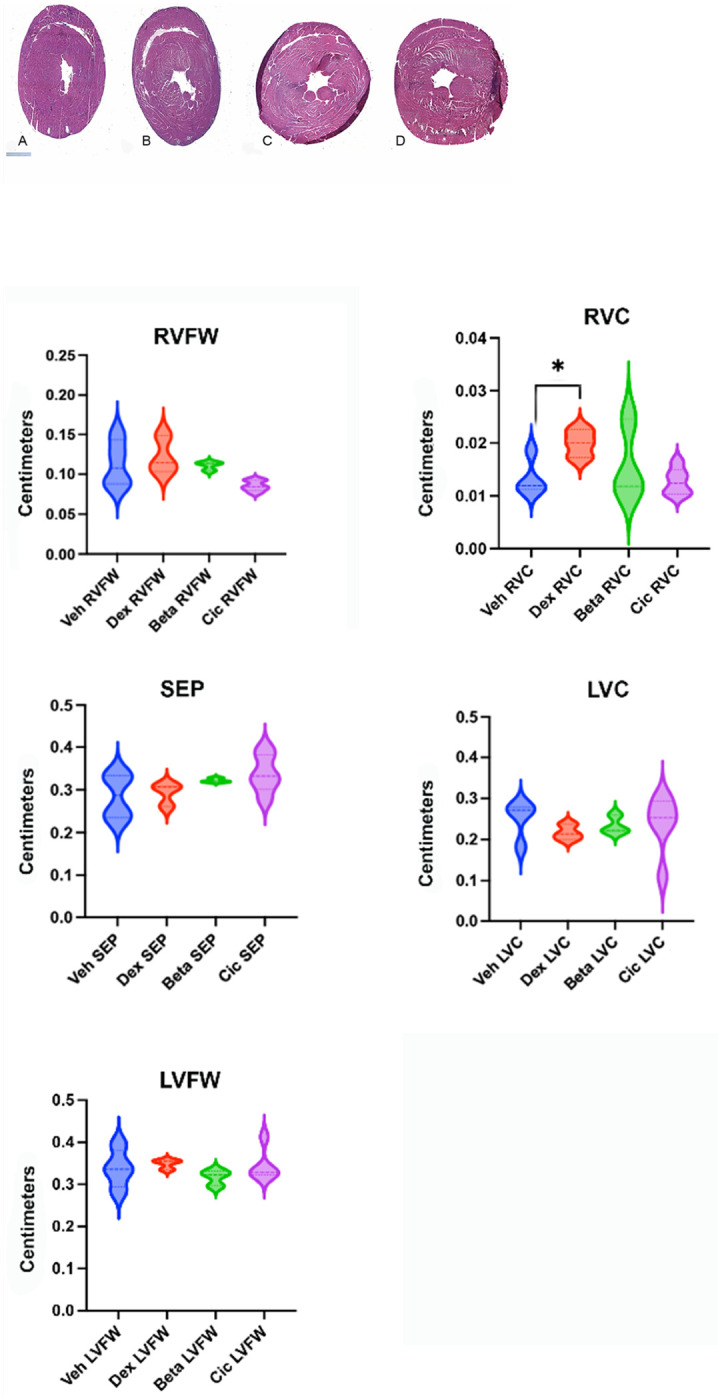
Histological and Structural Cardiac Alterations of Adult Mice Prenatally Exposed to Distinct sCS. Violin plots of Right Ventricular Free Wall (RVFW), Right Ventricular Chamber (RVC), Septum, Left Ventricular Chamber (LVC), and Left Ventricular Free Wall (LVFW) measurement comparisons in adult mice. Dex-treated mice had an approximate 50% increase in the size of the right ventricle compared to control (N=4; p = 0.043).

Previous studies highlighted cardiac alterations after repeated antenatal exposure to CS’s^41,45^. To determine whether a single exposure to antenatal CS’s led to cardiac abnormalities in adults, hearts were examined at 92 weeks. Heart wall and chamber sizes were measured, and structural analysis revealed a significant difference in heart size and chamber dimension in Dex-exposed animals versus controls. In adult animals, a marked increase in the proportional ratio of the right ventricular chamber to the diameter of the heart in the Dex-exposed groups was observed. Specifically, Dex-treated animals exhibited a 1.5-fold proportional increase (50% enlargement) in right ventricular chamber compared to controls (N = 4, p = 0.043). Beta- or Cic-exposed did not show statistical differences in right ventricular chamber size (N = 6 per treatment, p = 0.543 and p = 0.733 respectively, Fig. 4a–e) or any other measurement. These findings suggest that antenatal exposure to Dex, may lead to substantial alterations in right heart chamber size, reflecting the long-term effects of these drugs on cardiovascular development.

**Figure 5:**
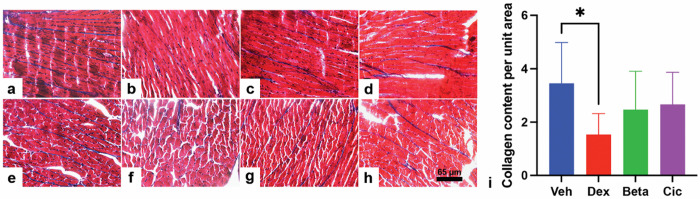
Cardiac Collagen Quantification and Analysis in Adult Mice Prenatally Exposed to Distinct sCS. a-h: Longitudinal (a-d) and transverse (e-h) 20x images of hearts stained with Masson-Trichrome to detect collagen in adult mice prenatally exposed to Veh (a,e), Dex (b,f), Beta (c,g), and Cic (d,h). Blue-stained collagen fibers were less abundant in Dex-exposed mice. Muscle fibers were stained red. 5I: Quantification of collagen content in sectioned adult hearts. A 56% decrease in connective tissue surface area was observed in Dex-exposed mice compared to control. (N=5, p = 0.034). Scale Bar= 65 μM

Alteration in muscle or extracellular matrix content can lead to changes in ventricular size. Collagen is one of the major structural proteins in the heart and is required to maintain the structural integrity of tissue, with its abnormal distribution associated with the onset of heart disease^49^. Previous studies have shown that Dex administration decreases collagen type IV synthesis in lung in postnatal animals^50^. To determine whether additional structural differences were present in sCS-exposed hearts, we examined cardiac collagen content. Trichrome staining revealed a significant reduction in cardiac collagen content in Dex-exposed animals, with a 56% decrease in collagen deposition compared to Veh-exposed (N = 5, Signal intensity/unit area = Vehicle 3.46 ± 0.62; Dex, 1.53 ± 0.36; p = 0.034). Statistical differences in collagen content were not observed in Beta-exposed hearts (N = 6, Signal intensity/unit area = 2.47 ± 0.36) or Cic-exposed hearts (N = 7, Signal intensity/unit area = 2.67 ± 0.45) (p = 0.348 and p = 0.205, respectively, Fig. 5). This reduction suggests that a single antenatal exposure to Dex leads to long-term changes in cardiac extracellular matrix, potentially compromising structural integrity and contributing to long-term cardiovascular dysfunction.

### Antenatal exposure to sCSs leads to distinct electrophysiological alterations and cardiac channel gene alterations in adults:

**Figure 6:**
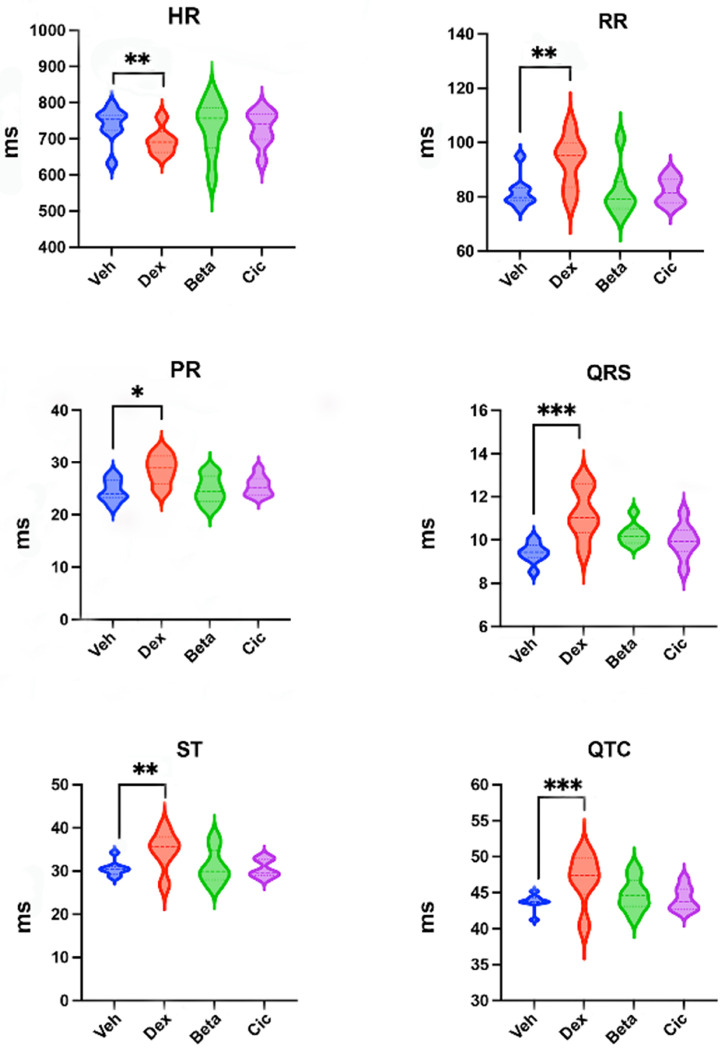
Electrocardiogram Analyses in Adult Mice Prenatally Exposed to Distinct sCS. Violin plots comparing electrocardiogram (ECG) recordings of adult mice prenatally exposed to sCS compared to control. Dex-exposed adult animals exhibited prolonged heart rate (HR; p = 0.006), RR (p = 0.0016), PR (p = 0.0273), QRS (p = 0.0001), ST (p = 0.002), and QTc (p = 0.0009) intervals compared to Veh (N=5–8 per treatment; 681 bpms vs. 754 bpms respectively). Beta- and Cic-exposed mice did not demonstrate significant changes in ECG intervals compared to control.

Considering the structural changes observed in the Dex hearts compared to Beta, Cic, or Veh, we assessed the functional impact of antenatal exposure to sCSs on heart function at 72–76 weeks of age. Electrocardiogram (ECG) recordings were conducted, and significant changes in electrophysiological parameters were observed (Fig. 6). Dex-exposed adult animals exhibited a decreased HR (Veh 754 bpms versus Dex 681 bpms, p = 0.006) with a prolonged RR (p = 0.0016), PR (P = 0.0273), QRS (p = 0.0001), ST (p = 0.002), and QTc (p = 0.0009) intervals compared to Veh, indicating altered cardiac conduction (N = 5–8 per treatment). Beta and Cic-exposed adults did not show any significant changes in electrophysiological parameters compared to Veh, suggesting minimal impact on conduction (Fig. 6).

**Figure 7:**
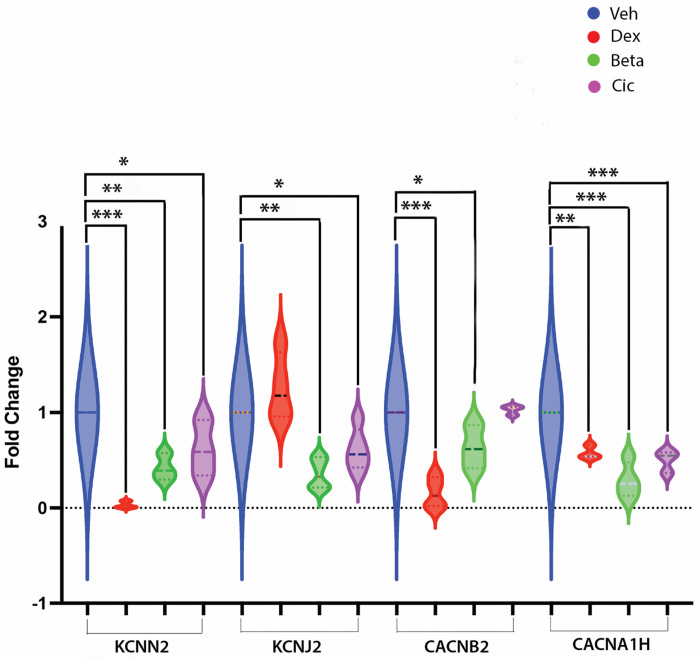
Quantitative Analysis of Selective Cardiac Ion Channel Gene Expression via qPCR Quantitative Polymerase Chain Reaction (qPCR) expression levels of several key genes encoding cardiac ion channels. Compared to control, Dex-exposed mice had significantly decreased expression of KCNN2 (N=6, p < 0.0001), CACNB2 (N=4, p < 0.0001), and CACNA1H (N=3, p < 0.0063). Beta-exposed mice had significantly decreased KCNN2 (N=3, p = 0.001), KCNJ2 (N=6, p = 0.001), CACNB2 (N=2, p = 0.031), and CACNA1H (N=4, p < 0.0001). Cic-exposed mice had significantly decreased KCNN2 (N=4, p = 0.016), KCNJ2 (N=5, p = 0.042), and CACNA1H (N=3, p = 0.002).

To gain an insight into potential molecular underpinnings of the electrophysical alterations induced by Dex, we examined the expression of cardiac ion channel genes that play central roles in regulating rhythmicity and contractility^51,52^. Cardiac depolarization/repolarizaiton and contraction/relaxation is regulated by conductance of sodium, potassium and calcium ion channels, we therefore performed quantitative polymerase chain reaction (qPCR) on tissue isolated from adult chamber walls to analyze the expression of select genes encoding cardiac ion channels. Dex-exposed animals exhibited a marked decrease in the expression of several channel genes examined. KCNN2 is a subtype of small-conductance calcium-activated potassium channels that plays a crucial role in the repolarization of cardiac cells. Its expression was reduced by approximately 99% with Dex, (N = 6, p < 0.0001), 58% with Beta (N = 3, p = 0.001), and 38% with Cic (N = 4, p = 0.016). KCNJ2, an inward rectifier potassium channel that helps to establish the resting membrane potential during repolarization, expression was reduced by 64% with Beta (N = 6, p = 0.001) and 39% with Cic (N = 5, p = 0.042); no significant difference with Dex (N = 4, p = 0.375) was observed. CACNB2, a subunit of L-type voltage-dependent calcium channels that plays a role in voltage sensitivity for activation and peak calcium entry for depolarization and contractility, decreased by 84% with Dex (N = 4, p < 0.0001) and by 37% with Beta (N = 3, p = 0.031); expression was unaltered with Cic (N = 3, p = 0.990). CACNA1H, a Cav3.2 T-type calcium channel found in pacemaker cells and contributes to pacemaker activity as well as ventricular cells and contributes to excitation contraction coupling, was reduced by 42% with Dex (N = 3, p = 0.0063), 71% with Beta (N = 4, p < 0.0001), and 50% with Cic (N = 3, p = 0.002) (Fig. 7). These results indicate that a single antenatal exposure to Dex, Beta or Cic leads to graded CS-specific alterations in the expression of genes implicated in regulating cardiac electrophysiological and functional parameters in adults.

## Discussion

sCS are extensively used to prevent adverse pulmonary, gastrointestinal and neurovascular complications associated with prematurity, however several studies have identified negative secondary consequences, with the pace of these studies accelerating dramatically over the last decade. Our study contributes to this growing body of research by focusing on aspects that remain largely unexplored. While most prior studies have concentrated on the antenatal administration of high-dose or repeated dose sCS exposure, effects of lower clinically relevant single antenatal doses have not been thoroughly investigated. This gap in research is particularly important given that clinical and translational studies have largely focused on the immediate effects of these drugs. However, the long-term consequences, especially in terms of cardiovascular health, also remain underexplored. While short-term studies have provided valuable insights, there is a pressing need for further research to uncover the full spectrum of long-term consequences associated with antenatal sCS exposure, particularly in areas like heart development, heart function, and metabolic health.

This study provides new insights into the long-term effects of antenatal sCS exposure on growth, cardiovascular health, and cardiac development in an animal model. Our findings align with and extend previous research, demonstrating that antenatal sCS exposure significantly impacts birth weight, growth trajectories, and cardiovascular function and electrophysiology. Notably, we observed both recovery and persistent alterations in various physiological parameters, which highlight the complexities of sCS exposure during pregnancy.

It is well-established that repeated antenatal administration of sCS leads to a significant reduction in birth weight of the offspring in both humans and rodent models^19,40–42^. Our data similarly confirms that a single antenatal exposure to sCSs significantly reduces birth weight. Specifically, Dex, Beta, and Cic exhibited lower birth weights compared to the Veh group. Interestingly, while all sCS-exposed groups showed reduced birth weights, Cic exposure led to a less pronounced weight reduction compared to Dex and Beta exposed, suggesting that Cic may be less impactful on fetal growth. Importantly, we have previously shown that repeated Cic administration in postnatal pups does not lead to the growth reduction observed with Dex exposure^34^. This observation may reflect differences in the route of exposure, potency, or pharmacological actions of these drugs *in utero*; this could have important implications for clinical treatment protocols, particularly in premature births.

Though the birth weight differences were significant, we observed full recovery in growth by week 5 with no differences between groups. This phenomenon is supported by research based on the ACTORDS randomized trial, which found that despite the initial reduction in weights at the time of birth, babies exposed to repeated sCS showed a postnatal growth acceleration 3–5 weeks after birth^42^. However, as animals aged, sCS-exposed pups exhibited significant increases in weight compared to controls. By week 92, Dex-exposed animals were significantly heavier than controls that was largely due to an increase in observed abdominal adipose content, Cic eposed were indistinguishable from controls, while Beta-exposed subjects showed a trend toward lower weight. These findings are interesting because of the significant role that both endogenous and exogenous corticosteroids play in lipid metabolism, promoting both lipogenic and lipolytic activities depending on context^53–57^. Studies in humans and animal models have also shown that repeated exposure to sCS in adults leads to fat deposition^58^, and antenatal exposure to sCSs or to factors that elevate maternal cortisol levels (such as maternal stress) are associated with an increased risk for metabolic disease in offspring later in life^59^. Both prolonged endogenous cortisol exposure or sCS exposure has been shown to lead to both acute and long-term epigenetic changes, such as histone modification and DNA methylation that can persist through generations^59–61^. The early effects of sCS exposure on growth may be mediated by epigenetic changes in-utero in genes that regulate lipid metabolism and may contribute to long-term alterations in body composition^44^. The lack of significant weight differences in the Cic exposed adults relative to controls indicates that Cic may differentially alter lipid metabolic targets, leading to minimal impact on long-term growth compared to Dex and Beta.

No differences in postprandial blood glucose, cholesterol levels, or survival rates were observed in any of the adult experimental groups compared to controls, despite the known associations between sCS exposure and metabolic disturbances like insulin resistance and hyperglycemia. This supports previous research, suggesting that lower dose sCS exposure has fewer metabolic effects than repeated or high-dose exposure^62^. However, our study’s assessment of metabolic parameters was limited to one timepoint in adulthood, and additional studies are needed to detect more subtle metabolic changes.

Corticosteroid signaling during fetal development is critical for structural and functional maturation of cardiomyocytes that depend on the timing and dose of administration^13^. While previous studies have shown that multiple and continuous antenatal doses of Dex in animals from E12.5 to E15.5 resulted in transient cardiac growth restriction^41^, our studies demonstrate that even a single antenatal dosage of Dex leads to alterations in cardiac morphology at birth. At P1, Dex-treated animals showed an increase in septal thickness associated with a decrease in left ventricle chamber diameter, indicative of hypertrophic cardiomyopathy with potential diastolic dysfunction at birth and in early periods of life. These findings are consistent with previous findings demonstrating that repeated antenatal Dex treatment transiently decreases the myocardial deceleration index, a marker of diastolic function at birth^63^. Interestingly, studies in both both animals and children exposed to sCS *in utero* exhibit transient hypertension at birth, possibly associated with the transient decreased ventricular chamber size observed in our studies in animals^64–71^. One notable limitation of this study is that echocardiograms were not performed in these mice, which could have provided an accurate assessment of ventricular function.

By 92 weeks of age, marked differences in heart structure between sCS exposures was observed that were distinct from findings at birth. Dex-exposed animals exhibited a 50% increase in size of the right ventricular chamber (RVC) and a 54% decrease in extracellular matrix collagen content in cardiac muscle compared to control, Beta-, or Cic-exposed. The decrease in collagen was not associated with a significant decrease in the LVC size, which could be due to anatomical differences as the left ventricular wall is larger than the right in healthy mice and humans^72,73^. The increase in the RVC in Dex-exposed animals suggests long-term alterations in cardiac structure that may be indicative of susceptibility to dilated cardiomyopathy. We cannot rule out that changes in the RVC were not due to primary alterations in pulmonary vasculature, which were not examined in this study. The observed alterations are consistent with findings from studies that link antenatal stress and corticosteroid exposure to alterations in cardiovascular development^58^. These findings are supported in human studies, which demonstrate that exposure to corticosteroids is linked to decreased fibroblast activity and decreased tissue collagen deposition in skin^74^. The identification of several corticosteroid receptor binding sites in genes implicated in regulating collagen further support these hypotheses^75–77^. The alteration of the extracellular matrix may contribute to the observed right ventricle chamber structural changes which could have functional implications for the heart’s susceptibility to chamber dilation, cardiomyopathy, and heart failure in adulthood. The lack of pronounced changes in heart structure in the Beta and Cic groups indicates limited long-term reprogramming of cardiac pathology.

Electrophysiological assessments revealed cardiac conduction deficits in Dex-exposed animals, including prolonged RR, QRS, ST, and QTc intervals, findings that are consistent with cardiac conductance changes. Specifically, prolonged RR interval indicates a lower resting heart rate as observed, possibly due to decreased excitability in the sinoatrial node pacemaker cells. Longer QRS complexes indicate prolonged atrial repolarization and ventricular depolarization, and prolonged corrected QT interval indicates longer time to repolarize the ventricles. Cic and Beta antenatal exposure did not lead to long-term differences in the ECG waveforms. Preliminary insight into the molecular underpinning of the ECG changes induced by Dex demonstrates changes in gene expression for specific cardiac ion channels. Dex, Beta, and Cic exposure led to decreases in expression of key potassium and calcium ion channel genes. These include KCNN2 that encodes a small-conductance calcium-activated potassium channel, essential for late-phase repolarization and hyperpolarization in cardiomyocytes and CACNA1H that encodes the α1H subunit of the T-type calcium channel required for pacemaker cell depolarization and ventricular physiology. KCNN2 is decreased in a graded manner for KCNN2 with Dex − 97% >Beta − 58%>Cic − 38%, and for CACNA1H with Dex − 42% >Beta − 71%>Cic − 50%, CACNB2’s expression is reduced by both Dex and Beta but not by Cic. The almost complete absence of KCNN2 (−96%) and CACNB2 (−84%) with the reduced CACNA1H (−42%) observed in adults exposed to Dex *in utero* compared to Beta and Cic may explain the dramatic changes in electrical conductance seen in the ECGs. Furthermore, these findings suggest that individuals exposed to Dex in utero may be at increased risk for arrhythmia’s, particularly bradyarrhythmia’s and Torsade’s de Pointes^78–82^.

Beta and Cic uniquely decreased KCNJ1, a mitochondrial ATP-sensitive potassium channel, in a graded manner with Beta − 64% > Cic − 39%. While functional ECG alterations were not observed with Beta or Cic exposure, a more detailed analysis may reveal other susceptibilities, for example in cardiac injury or fluid homeostasis^83^. While these studies have focused on the primary cardiac effects of prenatal sCS, the systemic effects of these drugs have not been examined. An important target of sCSs is the kidney, alterations in cardiac conduction may be due to renal electrolyte dysfunction. Interestingly, KCNJ1 inhibitors are currently being examined as therapeutic targets in heart failure to manage fluid retention^82^.

Concluding, our study provides insights into the immediate and long-term effects of a single clinically relevant antenatal sCS exposure. While we observed significant early developmental changes, including reduced birth weight and altered cardiac structure, these effects were not consistently linked to metabolic disturbances or survival outcomes in adulthood. Both Dex and Beta are used to reduce risks associated with perterm birth; of importance, they promote lung maturation and reduce the risk of respiripatory distress syndrome^12^. Cic has been shown to promte postnatal lung maturation in term born animals, both in an animal model of bronchopulmonary dysplasia and in an in-utero enterotoxin lung injury model^34,84^. This indicates that Cic comparatively activates similar pulmonary pathways. Despite the changes noted in cardiac gene expression with exposure to Cic, we noticed that these changes were much less profound compared to the electrophysiological gene profile implicated by Dex or Beta administration, suggesting that Cic may be a superior alternative for antenatal use in minimizing long-term health risks of premature children. While our studies focus on exposure to sCS in-utero, it is important to note that infants born preterm are also at significant risk for secondary pulmonary complications such as bronchopulmonary dysplasia. As noted in the DART trial, one treatment option for this pathology is the administration of tapering doses of Dex^85^, which has the potential to lead to additional cardiac pathological or conductance abnormalities. Interestingly, sinus bradycardia has been shown to be a common early side effect associated with prednisone treatment in children^52^. These findings highlight the importance of the antenatal environment in cardiovascular programming and suggest that individuals with known antenatal sCS exposure may benefit from long-term cardiac monitoring and risk stratification. The deficits observed can be consequence of a primary effect from sCS on distinct molecular targets, such as KCNN2^86^; or, they could be a secondary consequence of epigenetic modification induced in the microenvironment^60,61^. The identified molecular targets (KCNN2, CACNB2, CACNA1H) may represent potential therapeutic pathways for intervention in affected individuals. Notably, recent studies have linked the impact of premature birth itself to long-term cardiovascular alterations, possibly due to structural limitations imposed at birth^87^. Inherently, preterm birth may cause changes to both cardiac and vasculature structure and function, and it has been shown to specifically increase the long-term risk of cardiovascular disease, cardiometabolic disease, diabetes, hypertension, atrial fibrillation, and heart failure^88–92^. Ultimately, these studies highlight the need for continued research into the long-term consequences of antenatal sCS exposure, particularly regarding cardiovascular and electrophysiological health. Overall the results support the proposal that Cic may be a safer option for future use in managing preterm births than the current clinical regimen of Dex or Beta administration.

## Supplementary Material

Supplementary Files

This is a list of supplementary files associated with this preprint. Click to download.

- SupplementaryTables121.docx

## Data Availability

The data collected during experimental work performed for the purposes of this study are available from the corresponding author upon request.
